# Specialties

**Published:** 1880-01

**Authors:** J. J. Keyes


### SPECIALTIES.

J. J. KEYES.

A division of labor is more and more coming to be regarded both a necessity and an advantage. A necssity, because no one man can do everything ; an advantage, because one thing well followed is best done. A universal genius never achieves brilliant success *as* a universal genius. He may succeed moderately well in many things. Distinguished success results from patient and laborious application in one department of work. Most men possess a gift, an adaptation, which fits them for special tasks and definite spheres of action. An eminent New Testament writer said, long ago: "To one is given by the Spirit the word of wisdom ; to another the word of knowledge by the same Spirit ; to another faith by the same Spirit ; to another the gifts of healing by the same Spirit ; to another the working of miracles ; to another prophecy; to another divers kinds of tongues ; to another the interpretation of tongues." Recognizing this variety and difference of endowment, he also said : "Having these gifts differing according to the grace that is given to us, whether prophecy, let us prophesy according to the proportion of faith ; or ministry, let us wait on our ministering ; or he that teacheth, on teaching ; or he that exhorteth, on exhortation." Paul believed in specialties. And so did Jesus, as we see from his parable of the talents. Men's gifts, he meant to say, differ in kind and in proportion ; and all he asked was that every man should rightfully and faithfully use his "proper gift." Now, as men differ in their gifts, each man should first seek to know what his gift may be, and then select such sphere of action as that gift fits him to labor in the most naturally, easily and successfully. And even though a man may be so endowed and trained that he could succeed equally well in two or more professions, or kinds of business, he will, as a rule, do far better if he confines himself mainly to one. There is really no calling in life worth following which is not sufficient to engage all of any man's activity, or engross his whole attention. It is even true that many departments of labor are most successfully occupied when sub-divided, with one man or class of men engaged in each subdivision. Indeed, we see a necessity for the division of labor, and therefore of specialties, in every calling, and in every kind of business.

Take the mercantile business. In small country towns, where you find only one store, the merchant, of necessity, deals in almost every kind of goods : dry goods, groceries, hardware, drugs, patent medicines, geese feathers, dried apples, and things innumerable. But in cities it is found to be both necessary and convenient to run the mercantile business on the principle of specialties. The country merchant would like to do it, but he can't.

In the legal profession we observe the same tendency. Formerly the lawyer was "man of all work." Now we have our criminal lawyers, our civil lawyers, our office lawyers, our collecting lawyers, our pension lawyers ; that is, lawyers who select some one branch of legal practice, and give it their entire attention.

In literature we observe, also, the same thing. A Gibbon, a Hume, a Macauley, a Froude, a Prescott, a Motley, an Abbott, a Bancroft, having gifts developed by study and research, labors successfully in the department of history. A Dickens, a Bulwer, a Thackeray, a Scott, a Black, a George Eliot, having large imaginative and constructive faculties, cultured by education and use, excel in the department of fiction. A Chaucer, a Spenser, a Shakespeare, a Byron, a Burns, a Tennyson, a Longfellow, a Lowell, a Whittier, having large ideality and the power of interpreting nature, together with a profound knowledge of the human heart, becomes a poet. Any man's literary excellence will usually lie in one direction. You seldom find the historian, the novelist, the poet, the essayist, combined equally in the same person. The field is very large, and the specialist in literature is the man who achieves the truest excellence and the highest fame.

The field of *science*, also, is large, and furnishes splendid opportunities to the investigator, provided he does not undertake too much. The reason why Tyndall, Huxley, Darwin, Lyell, Cuvier, Le Monier, Herchel, Dana, Mitchell, and others have become so eminent as scientists, is that they did not any of them attempt to explore the entire field, but took a special branch of scientific inquiry, and gave it vigorous and untiring pursuit. Scientific truth may be a grand unit, but its ascertainment is by fractional parts by many investigators, who all supplement each other's work. The division of labor is needful because of the vastness of the field, and because of the diverse gifts, tastes and aptitudes of those who are engaged in its exploration.

If we look, also, at the department of medicine, we see, and we are coming to see more and more, the necessity and advantage of a division of labor. Indeed, this was seen by the Egyptians more than two thousand years ago. Herodotus wrote as follows : "The art of medicine in Egypt is thus exercised : One physician is confined to the study and management of one disease ; some attend to disorders of the eyes, others to those of the head ; some take care of the teeth, others are conversant with all diseases of the bowels ; whilst many attend to the cure of maladies which are less conspicuous." Time was, I think, when this practice among physicians must have become obsolete ; for I can remember the time, which is not even now I fear altogether gone by, when a specialist in medicine was regarded with suspicion, as though he were an innovator, and but little more to be trusted than a tyro, an amateur, or a quack. The man who, some years ago, advertised himself as a surgeon, an oculist, an aurist, a lung doctor, or set up for the special treatment of diseases of the blood, liver, kidneys or skin, was regarded by the "Regulars" with jealousy and disapprobation. A physician was supposed to know everything that needed knowing about disease, and to be thoroughly furnished for the treatment in all its forms and phases. But now it is getting to be understood both by the "profession" and by the people, that he who makes a special study of some particular branch of medical science (while of course he aims at a general knowledge of all), is most to be trusted for the treatment of those ailments that are included in his branch. And so it has come to pass that the man with a sick tooth goes for relief to the skilled dentist ; the man with a broken leg, to a professional surgeon ; the man with granulated eyelids, cataract, or any affection of the eye, to an oculist. And this is right. The doctor who makes any organ or disease of the body a subject of long and patient study, is supposed to know most about it, and is just the man to treat it.

The necessity and advantage of specialties, then, are apparent. Specialists, however, are exposed to two dangers, against which they should be on their guard ; they are constantly tempted to jealousy of each other, and they are liable to run in grooves. The specialist is a man with one idea. That idea he is accustomed to unduly exalt and magnify, until, at length, it fills his entire mental horizon. He sees nothing else. He thinks and talks of nothing else. He is apt to think his brother specialist has chosen a branch of study or labor far inferior to his own, and of much less value to the world. He needs to cultivate large-mindedness, and an interest in other men's pursuits and investigations, while at the same time he does not neglect his own. There should be, need be, no rivalry. No invidious emulation among men because they happen to be working in different departments of the world's great field. All have the same ends in view : the discovery of truth, and the prosperity, health and happiness of our common humanity; and these interests can be best subserved, in my view, by the method indicated in this article.

---

—Read every one of our excellent contributions. We start off in the new year with the very best number of the BISTOURY that has yet been produced. Do we not offer a most excellent magazine at very small cost; and is it not worth every cent we ask for it ?

